# Association of Serum Phosphate, Calcium and Alkaline Phosphatase With Risk of Incident Fractures in Healthy Older Adults

**DOI:** 10.1210/clinem/dgae099

**Published:** 2024-03-01

**Authors:** Sultana Monira Hussain, Ego Seeman, Hans G Schneider, Peter R Ebeling, Anna L Barker, Kevan Polkinghorne, Anne B Newman, Chenglong Yu, Paul Lacaze, Alice Owen, Cammie Tran, Mark R Nelson, Robyn Lorraine Woods, Bu B Yeap, David Clark, Lawrence J Beilin, John J McNeil

**Affiliations:** School of Public Health and Preventive Medicine, Monash University, Melbourne, VIC 3004, Australia; Department of Medical Education, Melbourne Medical School, The University of Melbourne, Parkville, VIC 3010, Australia; Department of Medical Education, Melbourne Medical School, The University of Melbourne, Parkville, VIC 3010, Australia; Alfred Health, Monash University, Melbourne, VIC 3004, Australia; School of Clinical Sciences, Monash University, Melbourne, VIC 3168, Australia; School of Public Health and Preventive Medicine, Monash University, Melbourne, VIC 3004, Australia; Silverchain, Melbourne, VIC 3000, Australia; School of Public Health and Preventive Medicine, Monash University, Melbourne, VIC 3004, Australia; Alfred Health, Monash University, Melbourne, VIC 3004, Australia; Center for Aging and Population Health, Department of Epidemiology, University of Pittsburgh, Pittsburgh, PA 15260, USA; School of Public Health and Preventive Medicine, Monash University, Melbourne, VIC 3004, Australia; School of Public Health and Preventive Medicine, Monash University, Melbourne, VIC 3004, Australia; School of Public Health and Preventive Medicine, Monash University, Melbourne, VIC 3004, Australia; School of Public Health and Preventive Medicine, Monash University, Melbourne, VIC 3004, Australia; Menzies Institute for Medical Research, University of Tasmania, Hobart, TAS 7000, Australia; School of Public Health and Preventive Medicine, Monash University, Melbourne, VIC 3004, Australia; School of Medicine, University of Western Australia, Perth, WA 6009, Australia; School of Public Health and Preventive Medicine, Monash University, Melbourne, VIC 3004, Australia; School of Medicine, University of Western Australia, Perth, WA 6009, Australia; School of Public Health and Preventive Medicine, Monash University, Melbourne, VIC 3004, Australia

**Keywords:** calcium, phosphate, alkaline phosphatase, fractures, older individuals

## Abstract

**Context:**

Aging increases fracture risk through bone loss and microarchitecture deterioration due to an age-related imbalance in bone resorption and formation during bone remodeling.

**Objective:**

We examined the associations between levels of phosphate, calcium (Ca), and alkaline phosphatase (ALP), and fracture risk in initially healthy older individuals.

**Methods:**

A post hoc analysis of the Aspirin in Reducing Events in the Elderly (ASPREE) trial recruited 16 703 Australian participants aged 70 years and older and 2411 US participants aged 65 years and older. Analyses were conducted on ASPREE-Fracture substudy participants from Australia with serum calcium, phosphate, and ALP measurement. Fracture data were collected post randomization. Cox regression was used to calculate hazard ratios (HRs) and 95% CIs. Phosphate, Ca, and ALP were analyzed in deciles (D1-D10), with deciles 4 to 7 (31%-70%) as the reference category. Restricted cubic spline curves were used to identify nonlinear associations.

**Results:**

Of the 9915 participants, 907 (9.2%) individuals had incident fractures recorded over 3.9 (SD 1.4) years. In the fully adjusted model, men in the top decile (D10) of phosphate had a 78% higher risk of incident fracture (HR 1.78; 95% CI, 1.25-2.54). No such association was observed for women (HR 1.09; 95% CI, 0.83-1.44). The population attributable fraction in men within the D10 phosphate category is 6.9%.

**Conclusion:**

This result confirms that high-normal serum phosphate levels are associated with increased fracture risk in older men.

Advancing age is accompanied by an increased risk of fragility fractures resulting from unbalanced bone remodeling (1). Over time this process leads to focal decreases in bone mass and a deterioration of bone microarchitecture. The accompanying bone fragility results in a lifetime risk of fractures that affects 1 in 3 women and 1 in 5 men (2). Remodeling involves resorption of calcium (Ca) and phosphate from bone, which may contribute to increased levels of these analytes. Concentrations of bone-related alkaline phosphatase (ALP) may also increase (1, 3, 4).

The possibility that increases in circulating Ca, phosphate, and ALP are associated with an increased fracture risk among older populations has been the subject of several investigation. Most studies from healthy populations have found no relationship between plasma Ca or ALP and the risk of fractures. However, a relationship between higher circulating phosphate levels and abnormal bone microarchitecture has been reported in patients with chronic kidney disease (CKD) (5). Moreover, 2 large population-based cohort studies—the Rotterdam and the Osteoporotic Fractures in Men (MrOS) studies—have reported that increased serum phosphate was associated with fracture risk in participants without CKD (6).

These findings are supported by evidence obtained from animal models in which increased dietary phosphate and bone fragility are reported even though circulating concentrations of phosphate remain within the normal range (7). We therefore aimed to examine the associations of phosphate, Ca, and ALP with fractures in an exclusively older population, known to be free of manifest cardiovascular disease or other life-threatening conditions, using the data from the Aspirin in Reducing Events in the Elderly (ASPREE). Unlike the Rotterdam and MrOS studies, the ASPREE participants were exclusively drawn from a healthy and older population.

## Materials and Methods

### Study Participants

The ASPREE recruited 16 703 Australians aged 70 years and older, and 2411 US participants aged 65 years and older between March 2010 and December 2014. Participants were community-dwelling older adults with no evident cardiovascular disease, dementia, physical disability, or chronic illness expected to limit survival to less than 5 years. Participants were randomly assigned to receive aspirin 100 mg daily or matching placebo (8). The ASPREE-Fracture substudy included the 16 703 Australian participants recruited to the ASPREE principal trial (9). Ethics approvals were obtained from the Monash University Human Research Ethics Committee (MUHREC) both for the ASPREE principal trial (2006/745MC) and the ASPREE-Fracture substudy (CF14/1740-2014000872). This study followed the Strengthening the Reporting of Observational Studies in Epidemiology (STROBE) reporting guidelines.

### Routine Assessments

Anthropometric and laboratory measurements were taken during yearly visits, while data on medical morbidities, chronic conditions, lifestyle, sociodemographic factors, prescription medications, and other relevant health variables were collected at the same time (10). The collection of data followed standard operating procedures for both visit conduct and individual test administration. Modified Fried's frailty phenotype, which defines frailty as the presence of weakness, slowness, exhaustion, low physical activity, and weight loss, was used to categorize frailty status. Participants with 1 or 2 criteria were categorized as prefrail, and 3 or more criteria were categorized as frail (11). We combined prefrail and frail because of the small numbers of frail participants within this cohort.

### Clinical Chemistry Data

A total of 12 011 Australian ASPREE participants provided nonfasting blood samples (n = 12 011) at or close to recruitment to the study. Serum aliquots were processed and stored at −80 °C in the ASPREE Biobank until measurement. The analyses were conducted in the clinical chemistry department at the Alfred Hospital using a dedicated Alinity ci instrument (Abbott Diagnostics). Serum phosphate and Ca were measured using a Roche COBAS Integra 800 automated analyzer. Phosphate detectable range was 0.0969 to 6.46 mmol/L, and Ca was 0.02 to 4.99 mmol/L.

For the present analyses, examining phosphate, Ca, and ALP, we included only participants for whom all biomarkers were measured. Those who were prescribed antiosteoporosis (anti-OP) medications, men having γ-glutamyl transferase greater than 62 U/L, and women having γ-glutamyl transferase greater than 38 U/L were excluded (the latter because of the likelihood that ALP levels in these individuals were likely to be increased as a result of liver pathology).

### Incident Fracture

The ASPREE-Fracture substudy collected data on fractures, including vertebral, hip, and other fractures, confirmed by medical imaging (radiographic imaging, magnetic resonance imaging, computed tomography, and bone scans) (9, 12). Information suggesting a possible fracture was collected during annual visits and 6-month telephone follow-ups, triggering collection of relevant information from hospitals, medical practices, and specialist records. An end point adjudication committee consisting of clinicians and research personnel, blinded to group allocation, reviewed the clinical data and only those cases with confirmatory evidence of fracture were included in the analysis. The majority of the study participants presented with a fracture after a fall from standing height (considered to be minimal trauma fractures) with the remainder falls on stairs, ladders, stools, etc (other trauma fractures).

## Statistical Analysis

For continuous variables the characteristics of the participants were compared using analysis of variance and for categorical variables using χ^2^ tests, according to the deciles of serum phosphate categories. Continuous variables were presented as mean (±SD); categorical variables were reported as frequency (proportion). The distributions of phosphate, Ca, and ALP were examined separately for men and women.

Cox regression was used to calculate the hazard ratios (HRs) and 95% CIs from the time of randomization to the first fracture occurrence during the ASPREE follow-up period. Phosphate, Ca, and ALP were analyzed according to deciles with D1 (lowest 10%) and D10 (highest 10%). Deciles 4 to 7 (31%-70%) were used as the reference category. Analytical models included adjustments for one or more of age, sex, body mass index (BMI), smoking status (current/former vs never), alcohol consumption (current vs never/former), moderate to high physical activity (longest walk without resting or walking capability >30 minutes), hypertension (HTN) (yes/no), diabetes (DM) (yes/no), dyslipidemia (yes/no), estimated glomerular filtration rate (eGFR), and random assignment to aspirin. Finally, phosphate, Ca, and ALP were coadjusted in the final model with the other variables. The associations between phosphate and incident fractures were also investigated using restricted cubic spline curves to determine whether there was evidence of a nonlinear association, treating a phosphate level of 1.13 mmol/L (median) as the reference.

Since rates of fractures are different in men and women (13), and levels of the serum analytes are different, we performed an interaction test between sex, and serum Ca, phosphate, and ALP.

To exclude a possible influence of CKD (results in high phosphate levels) (14), incident cancer (as a cause of fracture) (15) major adverse cardiovascular events (MACE) (16), major trauma fractures, and heavy alcohol drinking, we further restricted our analyses in participants without CKD (eGFR < 60 mL/min/1.73 m^2^), without incident cancer, without MACE, including only those with minimal trauma fractures and excluding participants who reported heavy alcohol drinking (>4 drinks in a day or >9 drinks per week). As part of supplementary analyses, we included participants taking anti-OP medications and participants with high levels of γ-glutamyl transferase (both previously excluded from main analyses).

The population attributable fraction (PAF) was calculated using the “punafcc” command in Stata, which implements the method recommended by Greenland and Drescher (17). The formula for PAF calculation used is ΣpF_i_[(HR_i_ − 1)/HR_i_], where pF_i_ is the proportion of fractures observed in the ith phosphate category and HR_i_ is the HR associated with that category (18).

Statistical significance was defined as a 2-sided *P* value less than .05. Statistical analyses were performed using Stata MP version 17 for Windows (StataCorp LP). Data analysis was performed from April 2023 to July 2023.

## Results

### Baseline Characteristics

Of 9915 participants with measures of serum phosphate, Ca, and ALP at baseline included in this analysis (Supplementary Fig. S1) (19), 907 (9.2%) had incident fractures (294 in men, 613 in women) during 3.9 (1.4 mean, SD) years. Serum phosphate, Ca, and ALP were normally distributed both in men and women (Supplementary Fig. S2) (19). Compared with those in D4 to D7 (31%-70%), participants in the top decile (D10) of phosphate had small but statistically significant increases in several markers of good health such as lower BMI, moderate to high physical activity (walking ability >30 minutes), and a lower prevalence of HTN, CKD, or DM (Table 1). No statistically significant difference was observed in the frequency of frailty/prefrailty, alcohol intake, or cigarette smoking.

**Table 1.** Baseline characteristics of ASPirin in Reducing Events in the Elderly trial participants overall, and by phosphate deciles

Characteristics	All	1-10th (D1)	11-20th (D2)	21-30th (D3)	31th -70th (D4-D7)	71-80th (D8)	81-90th (D9)	91-100th (D10)	*P*
N (%)	9915 (100)	1054 (10.6)	1061 (10.7)	979 (9.9)	3854 (38.9)	1120 (11.3)	872 (8·8)	975 (9·8)	
Age, mean (SD), y	74.9 (4.0)	74.6 (3.9)	74.7 (4.0)	74.9 (3.9)	74.9 (4.1)	74.8 (4.0)	74.9 (4.0)	74.6 (4.1)	.43
Female, n (%)	4941 (49.8)	520 (49.3)	514 (48.4)	455 (46.5)	1974 (51.2)	532 (47.5)	455 (52.2)	491 (50.4)	.05
Moderate to high physical activity, n (%)	6431 (64.9)	634 (60.2)	668 (63.0)	610 (62.3)	2524 (65.5)	747 (66.7)	600 (68.8)	648 (66.5)	.004
BMI, mean (SD)	28.0 (4.4)	29.1 (4.8)	28.6 (4.6)	28.3 (4.3)	27.9 (4.2)	27.5 (4.4)	27.4 (4.1)	27.0 (4.2)	<.001
Current/former smoking, n (%)	4437 (44.8)	484 (45.9)	487 (45.9)	428 (43.7)	1719 (44.6)	515 (46.0)	374 (42.9)	430 (44.1)	.93
Current alcohol use, n (%)	7936 (80.0)	807 (76.6)	840 (79.2)	786 (80.3)	3109 (80.7)	895 (82.2)	717 (82.2)	782 (80.2)	.10
Education, n (%)									.03
< 12 y of schooling	5863 (59.1)	664 (63.0)	651 (61.4)	592 (60.5)	2248 (58.3)	656 (58.6)	491 (56.3)	561 (57.5)	
≥ 12 y of schooling	4052 (40.9)	390 (37.0)	410 (38.6)	387 (39.5)	1606 (41.7)	464 (41.4)	381 (43.7)	414 (42.5)	
Hypertension, (%)	7316 (73.8)	830 (78.8)	815 (76.8)	738 (75.4)	2820 (73.2)	797 (73.1)	637 (73.1)	679 (69.6)	<.001
Chronic kidney disease (eGFR < 60), (%) (n = 9629)	1674 (17.4)	222 (21.5)	188 (18.3)	168 (17.5)	625 (16.7)	176 (16.2)	146 (17.2)	149 (15.7)	.01
Dyslipidemia, n (%)	6516 (65.7)	690 (65.5)	688 (64.8)	634 (64.8)	2508 (65.1)	750 (67.0)	594 (68.1)	653 (67.0)	.52
Diabetes, n (%)	915 (9.2)	123 (11.7)	115 (10.8)	93 (9.5)	345 (9.0)	94 (8.4)	69 (7.9)	76 (7.8)	.01
Prefrail/Frail (%)	3492 (35.2)	383 (36.3)	361 (34.0)	348 (35.6)	1322 (34.3)	407 (36.3)	319 (36.6)	352 (36.1)	.63
History of cancer, n (%) (n = 9878)	1966 (19.9)	219 (20.9)	214 (20.3)	202 (20.8)	738 (19.2)	223 (20.0)	173 (19.9)	197 (20.3)	.88
Incident cancer, n (%)	1487 (15.0)	151 (14.3)	155 (14.6)	138 (14.1)	610 (15.8)	151 (13.5)	131 (15.0)	151 (15.5)	.49
Incident MACE, n (%)	526 (5.3)	51 (5.8)	58 (5.5)	47 (4.8)	194 (5.0)	63 (5.6)	49 (5.6)	54 (5.5)	.91
Aspirin	4963 (50.1)	524 (49.7)	528 (49.8)	453 (46.3)	1962 (50.9)	579 (51.7)	454 (52.1)	463 (47.5)	.06
Calcium, mean (SD), mmol/L									
Men	2.35 (0.10)	2.35 (0.12)	2.34 (0.10)	2.34 (0.10)	2.35 (0.10)	2.35 (0.10)	2.34 (0.10)	2.36 (0.10)	.21
Women	2.37 (0.11)	2.39 (0.15)	2.37 (0.12)	2.37 (0.11)	2.37 (0.10)	2.37 (0.10)	2.36 (0.09)	2.35 (0.13)	.002
Phosphate, mean (SD), mmol/L									
Men	1.06 (0.15)	0.80 (0.06)	0.90 (0.02)	0.96 (0.01)	1.06 (0.04)	1.16 (0.02)	1.22 (0.02)	1.33 (0.07)	
Women	1.19 (0.15)	0.93 (0.06)	1.04 (0.02)	1.10 (.01)	1.19 (0.04)	1.29 (0.01)	1.34 (0.02)	1.45 (0.09)	
ALP (SD), U/L									
Men	77.1 (23.0)	78.8 (19.1)	78.6 (26.1)	76.5 (21.3)	76.9 (22.9)	76.4 (24.0)	77.7 (25.5)	75.7 (21.9)	.35
Women	80.9 (22.0)	82.8 (23.3)	84.1 (24.7)	80.3 (22.2)	80.5 (21.4)	80.5 (20.8)	80.4 (21.0)	78.4 (21.8)	.004

Abbreviations: ALP, alkaline phosphatase; BMI, body mass index; eGFR, estimated glomerular filtration rate; MACE, major adverse cardiovascular events.

### Sex and Serum Analytes

There was an interaction between sex and serum phosphate in fracture risk (*P* = .04); however, no interaction for serum Ca (*P* = .79) or ALP (*P* = .98) was observed.

### Serum Phosphate and Incident Fractures

**Figure 1.**
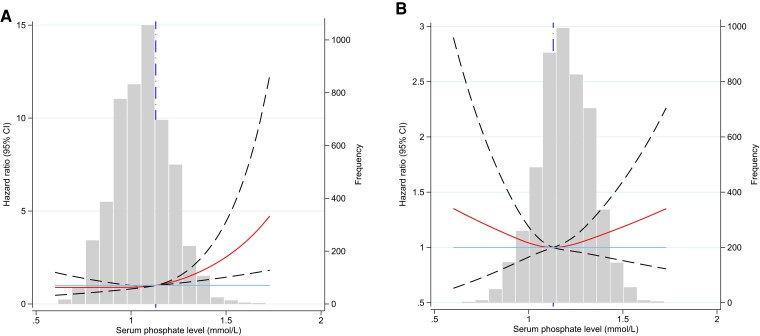
Spline curve to visualize the associations between serum phosphate and incident fracture in all population and in subgroups: A, men; B, women.

The associations between serum phosphate and incident fractures are presented in Table 2. After adjusting for age, BMI, smoking status, alcohol consumption, moderate to high physical activity, HTN, DM, eGFR, and randomization to aspirin (model 3), a 78% higher risk of incident fractures was observed among the men in the highest decile of phosphate compared to those in the middle deciles (D4-D7: 31%-70%). Coadjustment for serum Ca and ALP did not change these associations (model 4). No such association was observed for women. Fig. 1 presents the associations between serum phosphate and the risk of incident fractures using spline curves that demonstrate a linear upward trend in fracture risk with increasing phosphate levels, most pronounced in men. In women, the U-shaped relationship was less pronounced and did not reach statistical significance.

**Table 2.** Association between baseline serum phosphate and incident fractures (HR, 95% CI)

	Serum phosphate						
	1st-10th (D1)	11th-20th (D2)	21st-30th (D3)	31st-70th (D4-D7) (reference)	71st-80th (D8)	81st-90th (D9)	91st-100th (D10)
Male							
N (%)	24/534 (4.5)	36/547 (6.6)	21/524 (4.0)	110/1880 (5.6)	53/588 (6.0)	24/417 (5.8)	44/484 (9.1)
Risk/10 000 PY	110	164	101	147	150	146	243
Model 1	0.73 (0.47-1.13)	1.11 (0.76-1.62)	0.69 (0.43-1.09)	1	1.03 (0.70-1.51)	1.01 (0.65-1.57)	1.75 (1.23-2.48)
Model 2	0.74 (0.47-1.44)	1.14 (0.78-1.66)	0.69 (0.44-1.11)	1	1.05 (0.72-1.54)	1.02 (0.66-1.59)	1.76 (1.24-2.49)
Model 3	076 (0.49-1.18)	1.17 (0.80-1.71)	0.63 (0.39-1.03)	1	1.04 (0.70-1.52)	1.03 (0.66-1.62)	1.75 (1.23-2.49)
Model 4	0.76 (0.49-1.18)	1.16 (0.80-1.70)	0.63 (0.40- 1.03)	1	1.04 (0.71- 1.54)	1.04 (0.66- 1.63)	1.78 (1.25-2.54)
Female							
N (%)	67/520 (12.9)	49/514 (9.5)	57/455 (12.5)	257/1974 (13.0)	64/532 (12.0)	53/455 (11.7)	66/491 (13.4)
Risk/10 000 PY	328	237	324	335	307	300	347
Model 1	1.00 (0.76-1.31)	0.71 (0.52-0.96)	0.94 (0.71-1.26)	1	0.93 (0.71-1.23)	0.91 (0.68-1.22)	1.06 (0.81-1.39)
Model 2	1.01 (0.77-1.32)	0.70 (0.52-0.95)	0.95 (0.71-1.27)	1	0.92 (0.70-1.21)	0.90 (0.67-1.21)	1.08 (0.83-1.42)
Model 3	1.07 (0.82-1.40)	0.71 (0.52-0.96)	0.96 (0.72-1.28)	1	0.89 (0.67-1.18)	0.91 (0.67-1.22)	1.10 (0.83-1.44)
Model 4	1.07 (0.82-1.40)	0.71 (0.52-0.97)	0.96 (0.72-1.28)	1	0.89 (0.67-1.18)	0.91 (0.67-1.22)	1.09 (0.83-1.44)

Model 1: unadjusted. Model 2: adjusted for age. Model 3 adjusted for age, BMI, smoking status, alcohol status, physical activity, HTN, DM, dyslipidemia, eGFR, randomly assigned to aspirin. Model 4: model 3 coadjusted for calcium, phosphate, and ALP.

Abbreviations: ALP, alkaline phosphatase; BMI, body mass index; DM, diabetes mellitus; eGFR, estimated glomerular filtration rate; HR, hazard ratio; HTN, hypertension; PY, person-years.

Table 3 presents the relationship between phosphate and incident factures in men and women across several restricted groups. In the restricted analyses, results remained statistically significant among men free of cancer or MACE during the follow-up, among men without CKD (eGFR >60 mL/min/1.73 m^2^), among men who had minimal trauma fractures, and among men who did not report heavy drinking (see Table 3).

**Table 3.** Association between baseline serum phosphate and incident fractures based on sex, and analyses restricted to participants without chronic kidney disease, incident cancer, who had minimal trauma fractures, and excluding those who reported heavy alcohol drinking (HR, 95% CI)

	Serum phosphate						
	1st-10th (D1)	11th-20th (D2)	21st-30th (D3)	31st-70th (D4-D7) (reference)	71st-80th (D8)	81st-90th (D9)	91st-100th (D10)
Excluding chronic kidney disease*^a^* (n = 7736)							
Male	0.67 (0.40-1.10)	1.22 (0.82-1.82)	0.62 (0.36-1.05)	1	0.98 (0.64-1.48)	0.92 (0.56-1.53)	1.97 (1.36-2.84)
Female	1.01 (0.73-1.38)	0.64 (0.45-0.92)	0.97 (0.70-1.34)	1	0.97 (0.72-1.31)	0.89 (0.63-1.24)	1.18 (0.88-1.58)
Participants without incident cancer (n = 8138)*^b^*							
Male	1.17 (0.58-2.36)	1.79 (0.93-3.44)	1.68 (0.95-2.97)	1	1.53 (0.79-2.96)	1.76 (0.88-3.51)	3.51 (1.90-6.50)
Female	1.08 (0.74- 1.58)	0.72 (0.48-1.10)	1.05 (0.77-1.43)	1	0.90 (0.61-1.33)	0.99 (0.66-1.48)	1.07 (0.72-1.57)
Participants without incident MACE (n = 9068)*^b^*							
Male	1.15 (0.60-2.19)	1.75 (0.97-3.18)	1.58 (0.94-2.65)	1	1.72 (0.96-3.11)	1.62 (0.85-3.09)	2.72 (1.52-4.85)
Female	1.08 (0.75-1.55)	0.73 (0.49-1.08)	1.01 (0.75-1.35)	1	0.90 (0.62- 1.30)	0.92 (0.63-1.35)	1.12 (0.78-1.61)
Minimal trauma fracture (n = 8753)							
Male	0.77 (0.37-1.58)	1.11 (0.60-2.07)	0.71 (0.33-1.51)	1	0.85 (0.43-1.64)	0.99 (0.48-2.03)	2.46 (1.49-4.07)
Female	1.06 (0.71-1.60)	0.90 (0.59-1.36)	0.70 (0.42-1.15)	1	0.93 (0.62-1.41)	0.85 (0.54-1.33)	1.01 (0.66-1.54)
Excluding heavy drinkers (n = 9329)*^b^*							
Male	0.71 (0.44-1.13)	1.21 (0.82-1.79)	0.59 (0.35-1.00)	1	1.10 (0.75-1.64)	1.07 (0.67-1.70)	1.70 (1.17-2.47)
Female	1.08 (0.82-1.41)	0.72 (0.52-0.98)	0.95 (0.71-1.27)	1	0.89 (0.67-1.18)	0.91 (0.67-1.11)	1.11 (0.84-1.46)

Abbreviations: ALP, alkaline phosphatase; BMI, body mass index; DM, diabetes mellitus; eGFR, estimated glomerular filtration rate; HR, hazard ratio; HTN, hypertension; MACE, major adverse cardiovascular events.

^*a*^Adjusted for age, BMI, smoking status, alcohol status, physical activity, HTN, DM, dyslipidemia, randomly assigned to aspirin, and coadjusted for calcium, phosphate, and ALP.

^*b*^Adjusted for age, sex, BMI, smoking status, alcohol status, physical activity, HTN, DM, dyslipidemia, randomly assigned to aspirin, and coadjusted for calcium, phosphate, and ALP.

The PAR among those with serum phosphate in the highest decile was 6.9% (95% CI, 2.4%-11.3%) for men (Table 4). This means maintaining the serum phosphate level in men within the D1 to D9 category, as opposed to the D10 category, could potentially lead to 6.9% fewer incident fractures at the population level, particularly for individuals resembling this study population.

**Table 4.** Estimated population attributable fraction (%) of fractures if participants in the D10 phosphate category could maintain a phosphate level similar to the D1 to D9 category

	PAF (95% CI)	
	Male	Female
Model 1	6.9 (2.3-11.2)	1.2 (0-3.8)
Model 2	6.8 (2.3-11.1)	1.4 (0-4.1)
Model 3	6.9 (2.3-11.2)	1.4 (0-4.2)
Model 4	6.9 (2.4-11.3)	1.5 (0-4.2)

Model 1: unadjusted. Model 2: adjusted for age. Model 3 adjusted for age, BMI, smoking status, alcohol status, physical activity, HTN, DM, dyslipidemia, eGFR, and randomly assigned to aspirin. Model 4: model 3 coadjusted for calcium, phosphate, and ALP.

Abbreviations: ALP, alkaline phosphatase; eGFR, estimated glomerular filtration rate; BMI, body mass index; DM, diabetes mellitus; eGFR, estimated glomerular filtration rate; HTN, hypertension; PAF, population attributable fraction.

### Serum Calcium and Incident Fracture

As shown in Supplementary Table S1 (19), no statistically significant association between serum Ca categories and incident fracture was observed in either unadjusted and confounder-adjusted analyses. Furthermore, these findings remained consistent and statistically nonsignificant across different subgroups, that is, for men and women, participants who remained free of cancer or MACE, CKD, or those with minimal trauma fractures, and nonheavy drinkers (Supplementary Table S2) (19).

### Serum Alkaline Phosphatase and Incident Fracture

As summarized in Supplementary Tables S3 and S4 (19), no association was observed between serum ALP levels and incident fractures. Similarly, no statistically significant association was observed in restricted analyses.

Regarding inclusion of participants treated with anti-OP medications and participants with high γ-glutamyl transferase, when participants taking anti-OP medications were included, results remained similar with no statistically significant association between Ca and ALP levels and fracture risk. Higher levels of phosphate were associated with an increased risk of fractures in men (Supplementary Table S5) (19). The associations between phosphate, Ca, and ALP and risk of fractures also remained similar when we included the participants with high γ-glutamyl transferase (Supplementary Table S6) (19).

## Discussion

In community-dwelling healthy older men aged 70 years and older, higher serum phosphate levels were positively and significantly associated with incident fracture risk. The observed association was independent of age, BMI, smoking status, alcohol consumption, and other chronic conditions including HTN, type 2 DM, dyslipidemia, and CKD and remained significant after coadjusting for Ca and ALP. We did not detect a relationship between levels of Ca and ALP with incident fractures in male or female participants.

These analyses showed that increased fracture risk appeared only in the tenth decile of serum phosphate levels (91%-100%), suggesting that levels above a threshold of 1.33 mmol/L (SD 0.07) in men were associated with an increased risk of fractures. This observation is consistent with a report from the Rotterdam and the MrOS studies in which the thresholds were 1.1 mmol/L (6). These results indicate an increased fracture risk within the normal range of values of serum phosphate among men.

These results are similar to those of the Rotterdam and MrOS cohorts, in which the association between high phosphate levels and incident fractures was substantially stronger in men than in women (6). However, in that report the relationship in women was statistically significant (6). The relationship between higher serum phosphate levels and increased all-cause and cardiovascular mortality has also been reported among men but not women (20, 21). The disparity between men and women may reflect a difference in sensitivity to high phosphate levels, with women having higher average serum phosphate concentrations than men of similar age (3, 22, 23).

The United States–based National Health and Nutrition Examination Survey (NHANES) has reported that high dietary phosphate intake is associated with higher plasma phosphate levels (24) . However, no association between dietary phosphate intake and serum phosphate levels was detected in the MrOS study (6). Similarly, in ASPREE, the frequency of consuming foods containing high phosphate was not found to be associated with elevated serum phosphate levels (Supplementary Table S7) (19); nor was the consumption of these foods associated with incident fractures (Supplementary Table S8) (19).

Patients with cancer (25, 26) or MACE (3, 27) may be at risk for fractures or high serum phosphate but the association between increased levels of phosphate and higher incident fractures remained when the analyses were restricted to participants free of cancer or MACE.

The absence of an association between Ca levels and fracture incidence is consistent with a recent report involving younger participants from the UK Biobank (28). A cross-sectional study using data from the NHANES reported an association between high serum ALP and reduced bone mineral density (BMD) among individuals aged 20 to 40 years (29). Similarly, a hospital-based study from Taiwan reported an association between high serum ALP levels and a reduction in lumbar and femoral neck BMD among participants aged 22 to 89 years (30). Despite these findings, there was no relationship between ALP and fractures observed in this cohort. In the present study, the effect of Ca, phosphate, and ALP were assessed individually and in combination.

### Strength and Limitations

The results of this study are supported by a previous report linking higher serum phosphate levels to decreased BMD of the lumbar spine (6). The findings are primarily generalizable to healthy community-dwelling adults aged 70 years and older in whom fractures are common. The ASPREE-Fracture substudy employed intensive and rigorous data collection and expert adjudication of fractures based on radiology reports and radiographs (9, 12). Participants with abnormal liver function test were excluded. The study has the usual constraints of an observational analysis, although the internal consistency of the findings among key subgroups provides support for their validity. We could not perform an analysis restricted to hip fracture or vertebral fracture; however, we have restricted our analyses to minimal trauma fractures. The phosphate levels were measured in the nonfasting state, likely adding greater variability in the levels.

### Conclusions

These results provide confirmatory evidence that higher phosphate levels were independently associated with an increased risk of fractures in men as previously reported from the Rotterdam and the MrOS study. This association was not explained by serum Ca, ALP levels, or morbidities such as CKD, cancer, and MACE. The PAF for fractures in men within the D10 phosphate category is 6.9%. With fractures being prevalent among older individuals, further understanding of the mechanism underpinning this relationship could lead to interventions aimed at reducing the population burden of fractures.

## Data Availability

The data sets generated during the current study are available from the corresponding author with permission of the ASPREE principal investigators.

## Abbreviations

ALP: alkaline phosphatase
anti-OP: antiosteoporosis
ASPREE: ASPirin in Reducing Events in the Elderly
BMD: bone mineral density
BMI: body mass index
Ca: calcium
CKD: chronic kidney disease
D: decile
DM: diabetes mellitus
eGFR: estimated glomerular filtration rate
HR: hazard ratio
HTN: hypertension
MACE: major adverse cardiovascular events
MrOS: Osteoporotic Fractures in Men
PAF: population attributable fraction
